# Study on the Effect of Foam Stability on the Properties of Foamed Lightweight Soils

**DOI:** 10.3390/ma16186225

**Published:** 2023-09-15

**Authors:** Hao Liu, Cong Shen, Jixin Li, Gaoke Zhang, Yongsheng Wang, Huiwen Wan

**Affiliations:** 1 China Construction Second Engineering Bureau Limited East China Branch, Shanghai 200135, China; liuhao-2@cscec.com (H.L.); lijixin@cscec.com (J.L.); wangyongsheng_@cscec.com (Y.W.); 2 School of Materials Science and Engineering, Wuhan University of Technology, Wuhan 430070, China; shencong@whut.edu.cn (C.S.); gkzhang@whut.edu.cn (G.Z.)

**Keywords:** foamed lightweight soils, foam stability, pore structure

## Abstract

The properties of prepared foamed lightweight soils (FLSs) using prefabricated foam requires high foam stability. This paper investigates the geometrical characteristics of different foam densities, different types of foaming agents in the air, and the presence of slurry. Then, it studies their effects on the pore structure and mechanical properties of FLS. Results show that with the increase in foam density the bleeding rate of foam in the air for 1 h increases and the foam with a foam density of 50 kg/m^3^ is the most stable in the air. The stability of foam in slurry is not directly related to the property of foam in the air. The FLS prepared with the same foaming agent had the best performance with the FLS designed with a foam density of 50 kg/m^3^, which had the smallest average pore size and the most minor pore size distribution, and had the highest compressive strength. Among the three different foaming agents, Type-S was the best, and the slurry had the lowest rate of increase in wet density after the defoaming test, indicating that the foam had the best stability in the cement slurry. The FLS prepared with the density of 50 kg/m^3^ using the Type-S foaming agent and mixed with the slurry of cement, fly ash:slag:water = 105:105:140:227.5, was hardened to a mean pore size of 299 μm, and the 7 days, 28 days, and 56 days compressive strengths were 0.92 MPa, 2.04 MPa, and 2.48 MPa, respectively, which had the smallest average pore size and the highest compressive strength among the FLSs prepared using the three foaming agents.

## 1. Introduction

Foamed lightweight soil (FLS) is a lightweight cementitious material containing a large number of closed tiny pores formed by physically preparing an aqueous solution of a foaming agent into a foam, then mixing it with a cement slurry in a certain proportion, pouring it, and finally curing it naturally [1,2,3]. By controlling the amount of prefabricated foam, the density of the FLS can be between 300 and 1800 kg/m^3^ [4,5]. With its lightweight, good fluidity, ease of construction, high strength after hardening, and good integrity, FLS has been widely used in housing projects, railway and highway road foundations, military, civil engineering, airport runway end buffer paving, and other fields [6,7,8]. A large-scale test site for intelligent cars in Wuhan, China, has been successfully constructed using 860,000 m^3^ of FLS instead of ordinary fill as fill material for the road base on soft ground such as lakes and fishing ponds [9]. However, the disadvantages of FLS are also obvious: low compressive strength and poor stability. Moreover, as the density of the FLS becomes lighter, its compressive strength decreases, and its stability worsens [10]. Therefore, it is essential to develop FLS with high stability and strength [11].

The low stability of the prefabricated foam is the main reason for the instability of the FLS slurry [12,13]. It is generally believed that the better the foam stability, the better the performance of the FLS can be obtained [14]. Many researchers have conducted many studies on improving foam stability [15,16]. Liquid foam is a non-equilibrium system consisting of internal air and an external liquid film, and its evolution is influenced by three main mechanisms: foam drainage, film rupture, and bubble coarsening [17,18]. All three mean to separate the surfactant solution and the gas, allowing the liquid foam to eventually break into different surfactant solutions and gases to reach equilibrium. Krämer prepared particle-stabilized foams using nanoparticles and applied them to FLS [12]. They found that the mechanical properties of FLS designed with particle-stabilized prefabricated foams were superior to those with reference foams. Panesar investigated the effect of composite and protein foaming agents on the properties of FLS and concluded that composite foaming agents were selected for protein foaming agents [19]. In addition, Jones also pointed out that stabilizers have an important influence on the stability, density, and strength of the bubbles in the FLS and the workability and water retention of the slurry [10].

There is little research on the presence of foam in fresh slurry and the conditions under which it exists in fresh slurry. The foam in the air differs considerably from that in the slurry. In fact, the foam in the slurry is not only subject to frictional forces from the particles of the cementitious material but also interacts with them to produce adsorption effects [20,21]. Xu modeled the force on the foam in the slurry from the rheological properties of the slurry and decomposed the foaming process into four external forces. Wan studied the rheological parameters of the slurry, the internal foam situation in the mixing, and hardening stages to demonstrate that low-cementitious ratio FLS is feasible. Therefore, a stable foam in the air does not necessarily lead to preparing FLS with excellent properties [22]. Only good compatibility between the foam and the cementitious material composition will give the foam in FLS longer-term stability [23,24].

However, many studies only discuss the relationship between foam properties and FLS sample properties or discuss the relationship between FLS slurry and FLS sample properties. There is less research to analyze and investigate the combination of the three. There is no simple positive correlation between the three, and it is necessary to find the connection between them through systematic experiments. Therefore, this paper investigates the design and influence of foam stability on FLS prepared with different foam properties. The foam stability is quantified into two parameters: foam density and foaming agent types. The stability of foam in the air is investigated through the performance of foam prepared with two different parameters. Then, the influence of two parameters on the stability of FLS slurry and samples are explored through the defoaming test, pore structure, and compressive strength tests of FLS. The results of the above studies are combined to establish the relationship between the stability of foam in air and the properties of FLS.

## 2. Materials and Methods

### 2.1. Raw Materials

The cementitious materials used in this study to prepare the FLS were Portland cement (PC), fly ash (FA), and granulated blast furnace slag (GBFS).

The cement is P·O 42.5 cement produced by China Conch Cement Co., Ltd. (Wuhu, China) with the physical properties shown in Table 1. The FA is Class F Grade II produced by Hubei Koneng Environmental Protection Co. (Wuhan, China). The GBFS use grade S95 produced by Hubei Jinshenglan Metallurgical Technology Co. (Xianning, China). The chemical composition of raw materials is shown in Table 2.

Three foaming agents from different manufacturers were used in the test. The first is a ready-mixed compound reinforced foaming agent produced by Guangdong Shengrui Technology Co., Ltd. (Guangzhou, China) model JT-SRN2 (Type-S). The second is a compound foaming agent produced by Henan Huatai New Material Technology Co., Ltd. (Zhengzhou, China), model HTW-I (Type-H). The third is the mixed foaming agent produced by Wuhan Qianhesheng Building Material Development Co. (Wuhan, China) (Type-Q).

### 2.2. Specimen Preparation

This study is carried out on FLS for practical road-filling projects. The technical specifications of FLS must be 7 d compressive strength ≥ 0.5 MPa and 28 d compressive strength ≥ 1.0 MPa.

**Figure 1:**
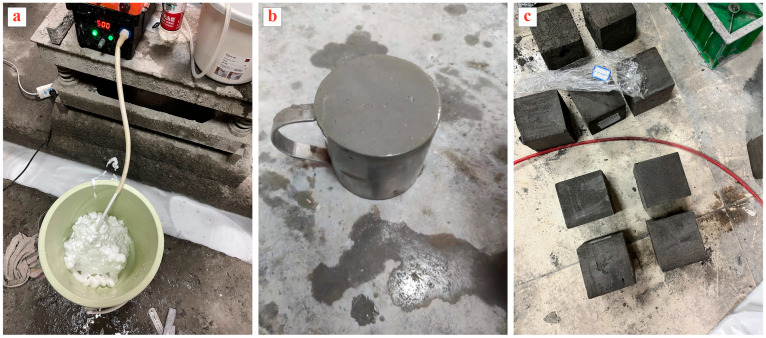
Preparation process of FLS: (**a**) Preparation of foam; (**b**) Preparation of FLS slurry; (**c**) Preparation of FLS sample.

In this paper, the preparation of FLS specimens was achieved using the prefabricated foam method. The preparation of FLS is shown in Figure 1. The designed wet density of FLS in the test was 600 kg/m^3^, and the water–binder ratio was 0.65 [25].

The process of preparing the FLS can be divided into the following steps:

1. Preparation of the foam: First, a certain amount of foaming agent is weighed. According to the dilution multiple of the foaming agent, a required amount of water is weighed. The weighed water is poured into a bucket and then the foaming agent is poured into the bucket and mixed into an aqueous foaming solution using a glass rod. Next, the switch of the intelligent micro foaming machine is turned on and the parameters of the foaming machine are adjusted. A uniform and even foam flow comes out of the foam outlet tube. Afterwards, the density of the foam is calibrated in a 1 L container and tested at 50 ± 2 kg/m^3^. Once the density of the foam meets the requirements, the foam is weighed in a drum using the mass method and set aside.
2. Preparation of the cementitious material slurry: The mixing water is weighed according to the designed ratio and divided equally into two parts. One is added directly to the bucket. The FA, GBFS, and PC are weighed and mixed according to the designed mix ratio, then added to the bucket containing one part of the water. During mixing, the other part of the water is poured into the bucket at a constant speed to make a slurry.
3. Preparation of the FLS: The prepared foam (in step 1) is added to the slurry (in step 2) at once, and then a mixer is used to mix the slurry. At the beginning of the mixing process, the mixer is placed on the upper surface so that the foam floating on the surface can dissolve in the slurry during the mixing processing and then move in a circular motion around the center of the barrel while oscillating up and down to ensure adequate mixing. After the slurry has been mixed evenly, the flow factor and wet density are measured, and the designed wet density of 600 kg/m^3^ is reached before shaping and pouring.
4. Casting and curing: The prepared slurry (in step 3) is poured into the prepared 100 × 100 × 100 mm^3^ triplex mold using a beaker. The mold is lightly vibrated at half its height. The final pouring is finished about 1–2 cm above the mold to prevent collapse when casting. The surface of the poured mold is then covered with a layer of cling film. Due to the low early strength of the FLS, demolding after 24 h of maintenance will result in incomplete specimens, so the finished specimens are stored for about 48 h before demolding. When demolding, the extra pouring part is scraped off with a scraper. After demolding, the samples are placed in a plastic bag and sealed before being placed in the curing room.

### 2.3. Mix Design

The performance of the foams in the experiments was characterized using two parameters, namely, the type of foaming agent and the foam density. The specimens prepared for the study of foam density were numbered SF40, SF50, and SF60, and those prepared for the study of foaming agent types were numbered HD60, QD80, and SD100, respectively. The specific mixes are shown in Table 3.

### 2.4. Test Methods

#### 2.4.1. Foam Performance Tests

The physical properties of the foam are tested for the 1 h settling distance and bleeding rate as well as the shape and diameter of the foam.

**Figure 2:**
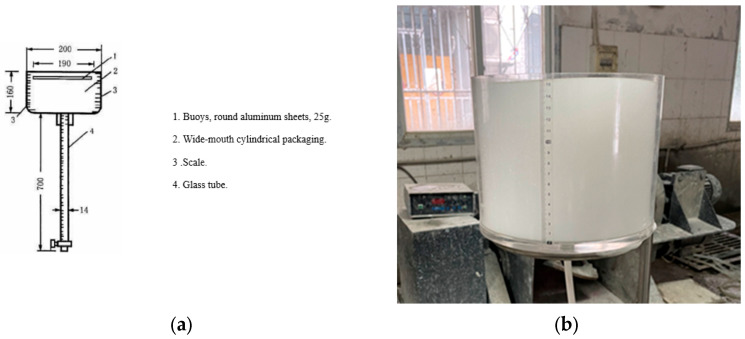
Shown is the 1 h settling distance and bleeding rate measuring instrument: (**a**) Instrument schematic; (**b**) Practical measurements.

The 1 h settling distance and bleeding rate of foams is carried out using a special measuring instrument. Figure 2 shows the measuring instrument.

The volume of the instrument is 5.015 L. After the foam has stood for 1 h, the settling distance of the buoy on the surface of the foam is first read through the scale on the wall of the vessel. Then, the foam liquid is passed through the glass tube in the lower part of the container, and its mass $m_{1h}$ is weighed. The bleeding rate of the foam for 1 h is calculated according to the following Equation (1):

(1) $$\varepsilon =\frac{m_{1h}}{\rho_{1}V_{1}}$$

where *ε* is the 1 h bleeding rate of the foam, in %; $m_{1h}$ is the mass of the foam flowing out after 1 h, in g; $\rho_{1}$ is the foam density, in g/mL; and $V_{1}$ is the volume of the container, which is 5.015 L.

The foam morphology and diameter were characterized using an optical microscope. A small amount of foam was prepared by aspirating it with a rubber-tipped dropper, then squeezing out a small amount onto a clean microscope slide, lightly covering the foam with a coverslip and placing it under the microscope. Adjust the magnification to 40× and observe the morphology and diameter of the foam.

#### 2.4.2. Stability Tests of Foam in Slurry

The stability of the foam with the slurry can be evaluated using the rate of increase in wet density of the FLS slurry after the defoaming test. Firstly, its initial wet density $\rho_{0}$ is determined using a 1 L volumetric bucket, and then it is stirred by hand at a slow speed (stirring speed 60 r/min) for 1 min. After each stirring, the density $\rho_{x}$ is measured. Repeating the stirring process 6 times, the maximum wet density $\rho_{6}$ is determined, and the rate of increase in wet density is calculated according to the following Equation (2):

(2) $$\delta =\frac{\rho_{6}-\rho_{0}}{\rho_{0}}\times 100\%$$

where δ is the rate of increase in wet density, in %; $\rho_{0}$ is the initial wet density of FLS, in g; and $\rho_{6}$ is the wet density of FLS after 6 mixes, in g.

#### 2.4.3. Pore Structure Tests

A body microscope was used to observe the pore structure of the hardened FLS. The test samples were first cut into 20 × 20 × 20 mm^3^ sizes using cutting equipment, and the surface was smoothed with sandpaper to expose the original pore structure inside the samples. The sample was then placed in the middle of the viewing area of a body microscope, and the focus was adjusted to observe the pore structure of the sample.

#### 2.4.4. Compressive Strength Tests

The compressive strength of FLS was tested according to standard GB/T 11969-2020 [26]. But, the samples were tested without water absorption or drying following standard CECS 249:2008 [27]. After the specimens reached the specified curing age, the samples’ apparent naturally dried densities (quasi-dry densities) were measured first. Then, the machine (TYE-300D) conducted the compressive strength test, and the loading speed was 0.1 kN/s.

## 3. Results

### 3.1. Stability of Foam in the Air

According to the characteristics of raw materials, synthesis process, and solids content, the manufacturer of the foaming agent will recommend the optimal dilution times when using it. In this paper, Type-S is recommended 100 times, Type-Q is recommended 80 times, and Type-H is recommended 60 times.

The stability of foam in the air was controlled by varying the type of foaming agent and the foam density by measuring the 1 h settling distance and 1 h bleeding rate of the foam and characterizing the particle size and morphology of the prepared foam by observing the prepared foam using an optical microscope to characterize the foam stability in air.

#### 3.1.1. Settling Distance and Bleeding Rate (1 h)

The results of the 1 h settling distance and 1 h bleeding rate of the three foaming agents used in the experiment under different foam density conditions are shown in Table 4.

The smaller the 1 h settling distance and bleeding rate, the more stable the foam is in the air and the lower the degree of foam drainage. From the test results, it can be found that as the foam density increases, there is a tendency for the 1 h settling distance and bleeding rate of each foaming agent to increase. This means that for the three foaming agents used in this test, the stability of foam in the air is all 40 kg/m^3^ > 50 kg/m^3^ > 60 kg/m^3^. The 1 h settling distance and bleeding rate vary for foaming agents at the same foam density, with foam Type-Q > Type-H > Type-S stability.

#### 3.1.2. Foam Density

**Figure 3:**
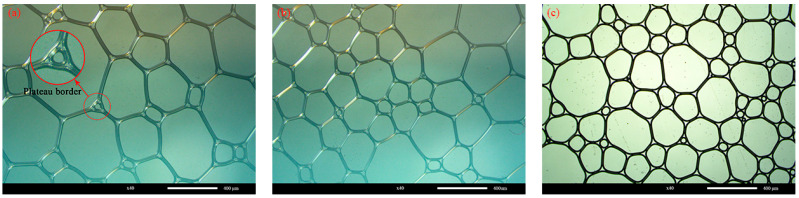
Foam morphology at different foam densities: (**a**) 50 kg/m^3^; (**b**) 60 kg/m^3^; (**c**) 40 kg/m^3^.

The morphology of the foams prepared with Types-S with densities of 40 kg/m^3^, 50 kg/m^3^, and 60 kg/m^3^ is shown in Figure 3 under an optical microscope.

It is apparent from Figure 3 that the shape of each group of foams is irregularly polygonal rather than spherical, as is commonly thought. This is because the foam density set in the experiments is relatively tiny. The prefabricated foams prepared using the foaming machine must be stacked to achieve the required foam density. When multiple foams are stacked together, the foams do not remain initially round and take on an irregular polygon shape. Moreover, it reaches its best stability when the number of sides reaches 6, which is determined by the draining effect of the foam film, the plateau boundary effect, as shown in the partial enlargement in Figure 3a.

**Figure 4:**
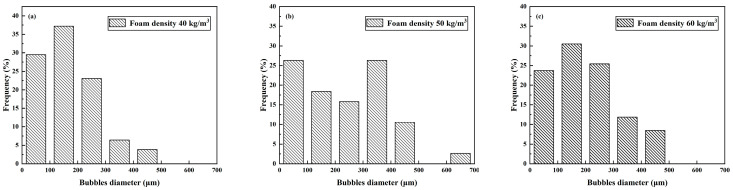
Foam particle size at different foam densities: (**a**) 40 kg/m^3^; (**b**) 50 kg/m^3^; (**c**) 60 kg/m^3^.

The foam morphology in the figure was measured and counted using ImageJ (https://imagej.net/ij/), and the results of the particle size distribution of the foam are shown in Figure 4. The results of the average diameter of the foam and the average thickness of the foam film are shown in Table 5.

As can be seen in Figure 4 and Table 5, the average diameter of the foam with a foam density of 50 kg/m^3^ is 236.28 μm larger than that of the foam with a foam density of 40 kg/m^3^ at 167.12 μm and 60 kg/m^3^ at 199.21 μm, which is contrary to conventional knowledge. The foam with a foam density of 50 kg/m^3^ has a more extensive diameter distribution. This indicates that the diameter size of prefabricated foams prepared using foaming machines does not have a simple linear relationship with density. Instead, there is a maximum value, and when the foam density is greater or less than this maximum value the diameter of the prepared foam becomes smaller. In addition, the average film thickness of 38.59 μm for a foam with a density of 50 kg/m^3^ is also more significant than the film thickness of 22.99 μm for a foam with a density of 40 kg/m^3^ and 23.62 μm for a foam with a density of 60 kg/m^3^. The increase in foam density from 40 kg/m^3^ to 50 kg/m^3^ is mainly due to the rise in liquid film thickness, while the increase in foam density from 50 kg/m^3^ to 60 kg/m^3^ is mainly due to the decrease in individual foam diameter and thickness and the increase in overall liquid film mass to increase the density. Therefore, for foam densities between 40 kg/m^3^ and 60 kg/m^3^ the diameter of the prepared foam increases and then decreases and the average liquid film thickness of the foam increases and then decreases.

#### 3.1.3. Foaming Agent Types

**Figure 5:**
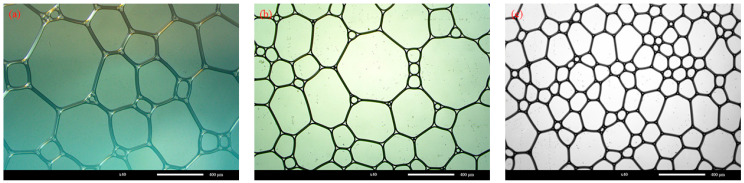
Foam morphology with different foaming agents: (**a**) SD100; (**b**) HD60; (**c**) QD80.

The morphology of the foams prepared using three foaming agents with a foam density of 50 kg/m^3^ under an optical microscope is shown in Figure 5. It can be seen that the morphology of the foams prepared by Type-Q and Type-H is similar to that of Type-S, which is also irregularly polygonal. Furthermore, although the foam densities of all three agents are 50 kg/m^3^, it is clear that their foam diameters and liquid film thicknesses are different.

**Figure 6:**
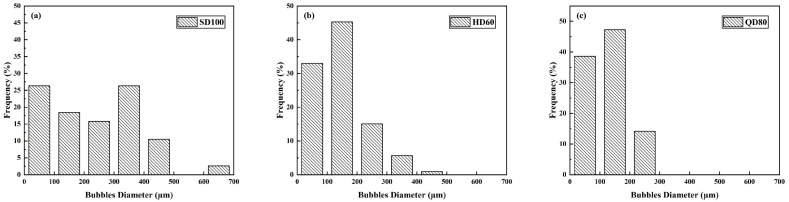
Foam particle sizes with different foaming agents: (**a**) SD100; (**b**) HD60; (**c**) QD80.

Similarly, ImageJ was used to measure and count the foam morphology. The particle size distribution of the foam is shown in Figure 6, and the results of the foam’s average diameter and the foam film’s average thickness are shown in Table 6. Figure 6 and Table 6 show that the average pore size of the foam prepared in the QD80 group was the smallest at 121.75 μm, with a small range of foam diameter distribution. The average pore size of the foam prepared in the HD60 group was 147.69 μm, again more minor than that of the SD100 group. In addition, the QD80 and HD60 groups’ average liquid film thicknesses were similar, around 22 μm. The small average diameter of the foam in the QD80 group and the small average liquid film thickness proves that the foam prepared in the QD80 group has the best stability in the air.

### 3.2. Stability of Foam in Slurry

The stability of the foam in the slurry system can be evaluated using the rate of increase of wet density after the defoaming test. Table 7 and Table 8 shows the rate of increase in wet density after the defoaming test of FLS prepared with different foam density and types of foaming agents. The lower rate of increase in wet density indicates that the foam is less likely to burst in the slurry system, and the stability is better. The rate of increase in wet density in practical engineering applications is generally controlled at less than 10%.

As seen from Table 7, for the same foaming agent, SF50 has the lowest rate of increase in wet density, which indicates that the foam film formed by the foam is thicker, so the foam is not easy to break and has good stability after repeated stirring. Table 8 shows that under the condition of the same foam density, sample SD100 has the lowest rate of increase in wet density. This result differs from the stability of foam in the air in Table 5. This indicates that foams in the air are not necessarily stable in slurry. It is not possible to directly use the performance of the foam in the air to determine its performance in the slurry.

### 3.3. Pore Structure of FLS

#### 3.3.1. Effect of Foam Density on Pore Structure

**Figure 7:**
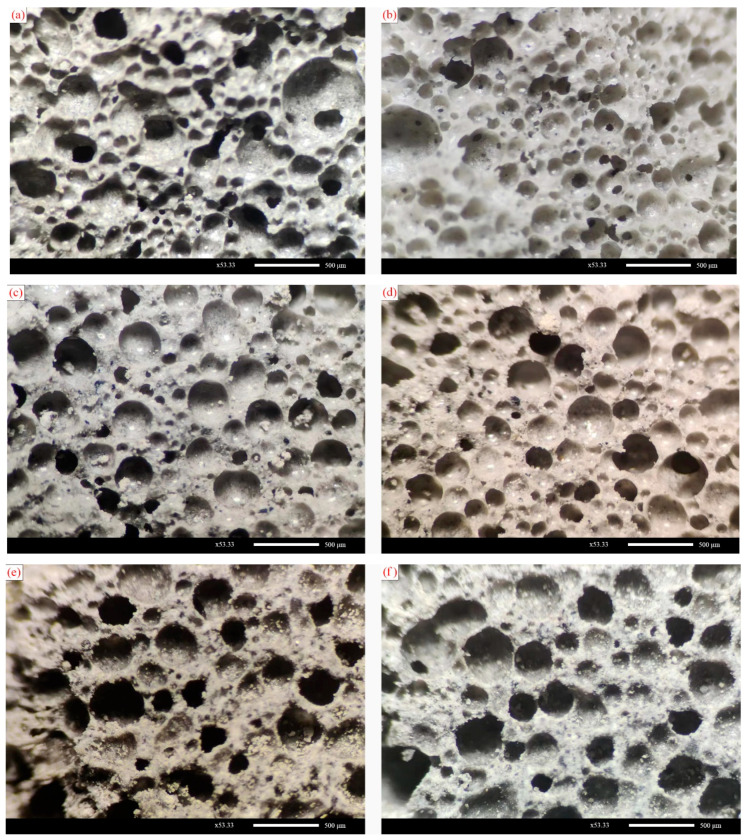
Pore structure diagram for different groups of FLS: (**a**,**b**) SF40 group; (**c**,**d**) SF50 group; (**e**,**f**) SF60 group.

To investigate the effect of foam properties on the microstructure of FLS after hardening, samples of SF40, SF50, and SF60 at 28 d hydration age were analyzed using a body microscope. The pore structure diagrams with a magnification of 53.33 times are shown in Figure 7.

As seen in Figure 7, the hardened FLS specimens, provided the pore morphology is not polygonal, are instead mostly round. This is not the same state as the foam morphology in the air. This is because, in a design for wet density of 600 kg/m^3^ FLS slurry, the volume share of the foam is theoretically between 60–70%. Suppose the foam is evenly dispersed around the cementitious material during the slurry mixing, forming the most compactly stacked structure. In that case, its volume share is approximately 78% more significant than the foam’s theoretical volume share. Therefore, foams that have built up on each other in the air will regain the round shape of the foam itself when they enter the FLS slurry. In addition, it can be found that the SF50 group has a more homogeneous pore structure, and the pores are mainly regular in shape; the SF60 group has larger pores and more pores have undergone consolidation as well as irregularly shaped pores, while the SF40 group has formed more small-grained pores. There are also many large pores with larger grain sizes and there is more connectivity between the hardened pores.

**Figure 8:**
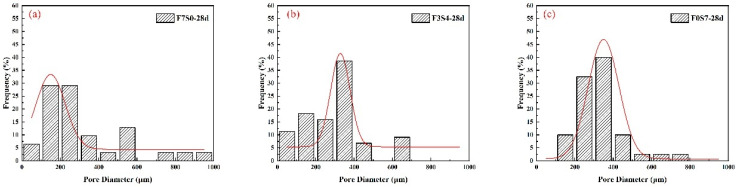
Pore size distribution of different groups of FLS: (**a**) SF40 group; (**b**) SF50 group; (**c**) SF60 group.

ImageJ was used to count the pore structures in the graphs. Trainable Weka Segmentation v3.3.2 was first used to identify the holes in the images, then binarized, and finally, the pore size distribution was measured for the processed images. In general, the pore size distribution of FLS obeys a normal distribution, and the pore size distribution is fitted using the normal distribution function Gaussian with the following fitting equation:

(3) $$f\left(D\right)=\frac{1}{\sqrt{2\sigma^{2}\pi }}e^{\left(-\frac{\left(D-\mu \right)}{2\sigma^{2}}\right)}$$

where $f\left(D\right)$ is the normal distribution probability function; *D* is the average diameter; *μ* is the mean deviation, and *σ* is the standard deviation. The standard deviation *σ* is related to the range of the pore size distribution, and the larger the value of its standard deviation *σ*, the more comprehensive the range of the pore size distribution. The fitted curves of the normal distribution of the pore size of FLS are shown in Figure 8. The statistical results are shown in Table 9.

**Figure 9:**
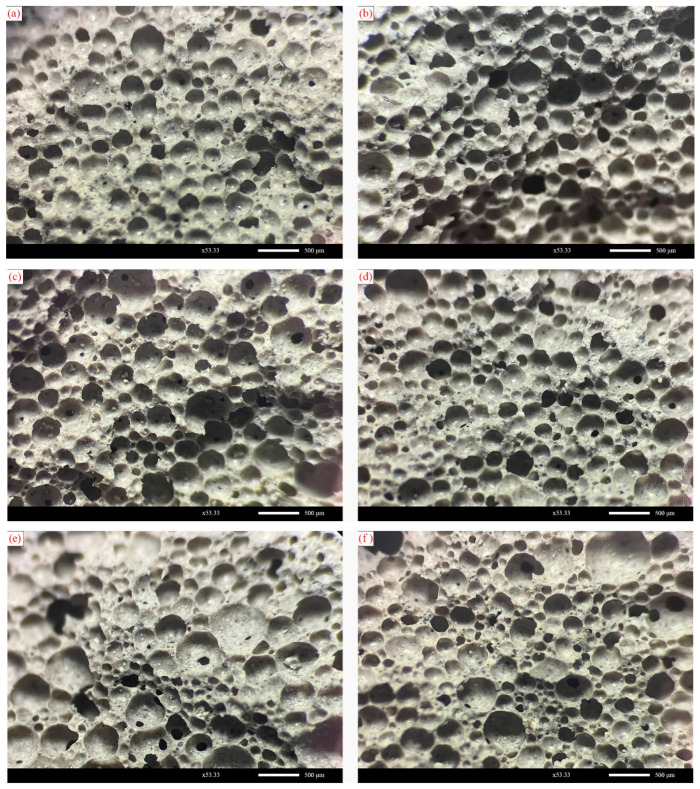
Pore structure diagrams of foam lightweight soils with different foam agents: (**a**,**b**) SD100 group; (**c**,**d**) HD60 group; (**e**,**f**) QD80 group.

As shown in Figure 9, the pore size distribution of the SF40 group shows a more significant proportion of tiny pores (<200 μm), accounting for approximately 34.4%. However, as it has a similarly large proportion of large pores (>700 μm), accounting for about 9.6%, the average pore size of the SF40 group is more prominent, at 365.63 μm, and the pore size distribution is vast, with a significant standard deviation of 172. This indicates that the pore structure formed by the small pore size foams is also smaller after hardening, but some of the small foams will merge, resulting in more large foams and large pores. The pore size distribution of the SF60 group shows a higher proportion of medium pores (200–400 μm), accounting for 72.5% of the pores, with an average pore size of 324.52 μm and a standard deviation of 131. Therefore, the average pore size of the SF50 group is the smallest. This suggests that more of the SF60 group’s foam was consolidated or destroyed during the forming and hardening. In summary, it can be seen that the SF50 group has the most minor pore structure and the best pore size distribution.

#### 3.3.2. Effect of Foaming Agent Types on the Pore Structure

As can be observed in Figure 9, the structure of the post-hardening holes in the SD100 and HD60 groups are primarily independent and do not have many interconnected holes. The QD80 group, on the other hand, has partially interconnected holes after hardening, and there are also cases where the larger holes contain smaller holes. However, overall, the pore structure formed after hardening in the SD100, HD60, and QD80 groups is circular. This indicates that foaming agent types does not excessively influence the shape of the pores after hardening.

**Figure 10:**
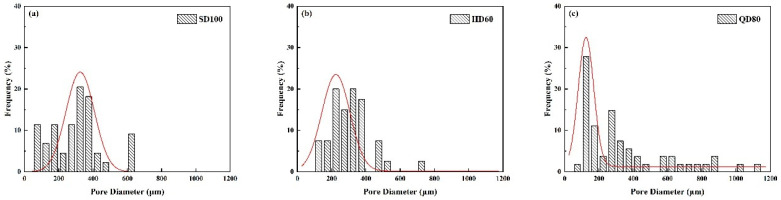
Pore size distribution of FLS with different foaming agents: (**a**) SD100 group; (**b**) HD60 group; (**c**) QD80 group.

ImageJ was used to count the pore structures in the figures, and finally, the pore size distribution was measured on the processed images; the results are shown in Figure 10. A standard distribution curve was fitted to the statistical aperture distribution and the results were obtained, as shown in Table 10.

As can be seen from Figure 10 and Table 10, the pore size distribution of the QD80 group shows that the QD80 group has a high proportion of tiny pores (<200 μm), accounting for approximately 42.4%. The pore size distribution of the SD100 and HD60 groups is similar, with the majority of pores between 200 and 400 μm in diameter, with an average pore size of 299.37 μm and 307.37 μm, respectively. The average pore size was 299.37 μm and 307.38 μm, respectively, and their standard deviations were around 100. This indicates that the pores formed after the hardening of the foam in the SD100 and HD60 groups were similar in size and distribution. However, the foam diameter in the air for the QD80 and HD60 groups is about 140 μm, whereas the foam diameter in the air for the SD100 group is 236 μm. It can be observed that there is no direct linear correlation between the size of the pores formed by the foam after slurry hardening and the diameter of the foam in the air. This indicates that the size of the foam in the air cannot simply be used to determine the pore size of FLS after hardening.

### 3.4. Compressive Strength of FLS

#### 3.4.1. Effect of Foam Density on the Compressive Strength

**Figure 11:**
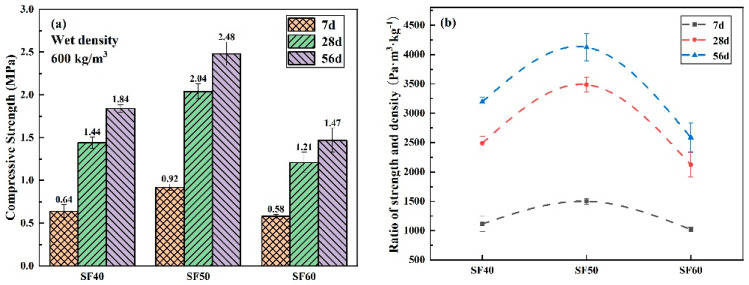
Strength of FLS at different foam densities: (**a**) compressive strength; (**b**) compression-to-density ratio.

The 7 days, 28 days, and 56 days compressive strengths and compaction-to-density of FLS prepared with Type-S at foam densities of 40 kg/m^3^, 50 kg/m^3^, and 60 kg/m^3^ are shown in Figure 11. As shown in Figure 11a, the compressive strength of FLS specimens tends to increase and then decrease with increasing foam density. Moreover, the compressive strengths at different ages are higher for the foam density of 50 kg/m^3^ than for the foam density of 40 kg/m^3^ than for the foam density of 60 kg/m^3^. This indicates that the compressive strength is not necessarily higher for FLS with a wet density of around 600 kg/m^3^, designed for a high theoretical wet density. The main influencing factors for compressive strength are the cementitious material’s composition and the incorporated foam’s properties. As can be seen from Figure 11b, similar to the effect of dilution multiplier on the compressive density ratio of foam lightweight soils, the increase between ages for specimens with different foam densities is about 150% from 7 days to 28 days and about 30% from 28 days to 56 days, which is not a significant difference. Again, it can be demonstrated that for the strength development of FLS, the cementitious material’s composition plays the primary role, independent of the foam density of the foaming agent itself.

#### 3.4.2. Effect of Foaming Agent Types on the Compressive Strength

**Figure 12:**
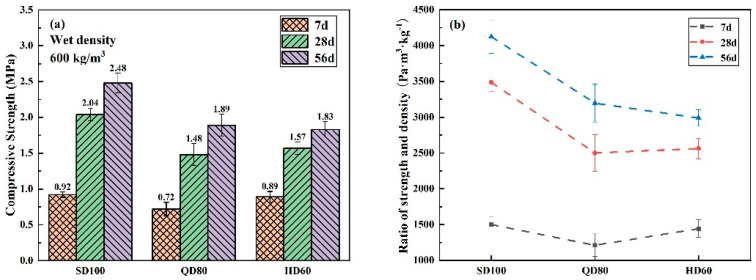
Strength of foam lightweight soils with different types of foaming agents: (**a**) compressive strength; (**b**) compression-to-density ratio.

The 7 days, 28 days, and 56 days compressive strengths and compaction-to-density of FLS prepared with the three foam agents are shown in Figure 12. Figure 12a indicates that the Type-S group has the highest compressive strength at all ages, with compressive strengths of 0.92 MPa, 2.04 MPa, and 2.48 MPa at 7 d, 28 d, and 56 d, respectively. The result can be better explained by the size and distribution of the pore structure of FLS in the previous section of 3.3.2. And, as can be seen in Figure 12b, the rise in the Type-Q and Type-H groups from 7d to 28d is only 105% and 78%, which is lower than the 130% of the Type-S group at the same stage. This suggests that foaming agent types can affect strength development similarly. This may be related to some substances added to the foaming agent. As the substances contained within each foam agent were not identified in the study, it can only be speculated that the foam agent used in the Type-S may have been mixed with ingredients with a strength-enhancing effect.

### 3.5. Analysis of Foam Properties on the Role of FLS

#### 3.5.1. Mechanism of Action of Foam Density

**Figure 13:**
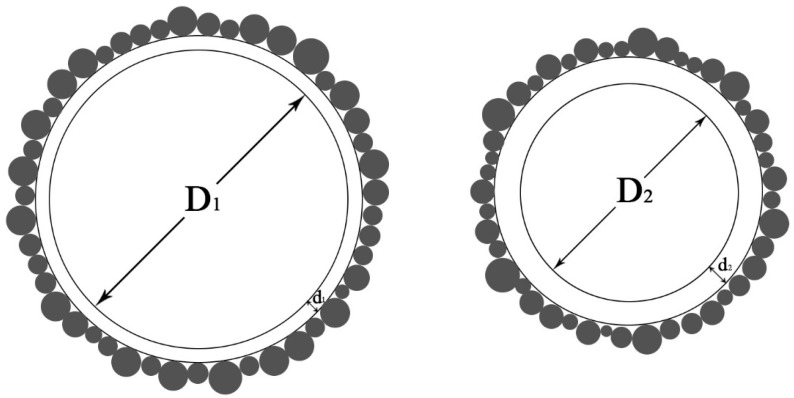
Presence of foam of different particle sizes in the slurry.

As can be summarized in the experiments and discussions in Section 3.1, the effect of foam density on foam properties is mainly achieved by influencing the prepared foam’s average diameter and the foam film’s thickness. For foam density, the foam’s average diameter and the foam film’s thickness tend to increase and then decrease at foam densities between 40 and 60 kg/m^3^. According to a particular form of the Young–Laplace equation: Equation (4) shows that the pressure difference on the foam in the slurry is proportional to the surface tension of the liquid film and inversely proportional to the radius of the foam so that the larger the radius, the more stable the foam is, irrespective of other factors.

(4) $$\Delta p=\frac{2\gamma }{R}$$

where Δp is the pressure difference to which the foam is subjected, *γ* is the surface tension of the foam film, and *R* is the foam’s curvature radius, in this case, the foam radius. During the mixing of the foam with the slurry, particles of gelling material are adsorbed on the surface of the foam film, and these particles attached to the foam surface reduce the free energy of the specific surface, thus reducing the surface tension. Under the combined influence of these two factors, within a certain range (less than the critical foam diameter) foams with a larger average foam diameter and a thicker foam film are more stable in FLS slurry, as shown in Figure 13.

For FLS with a designed wet density of 600 kg/m^3^, during the mixing and forming process, the foam first reverts from a stacked irregular polygon to a round shape after entering the slurry. Then, it adsorbs in contact with the cementitious material in the slurry, causing the cementitious material particles to adhere around the foam. During this process, the smaller radius foams or foams with a thinner film will merge and break up due to the foam’s drainage action, leaving behind stable foams in the slurry. Finally, these foams form holes during the hardening process. Therefore, for the conclusion of foam density on FLS, it can be concluded that for FLS with a design wet density of 600 kg/m^3^ a foam density of 50 kg/m^3^ gives better performance.

#### 3.5.2. Mechanism of Action of Foaming Agent Types

**Figure 14:**
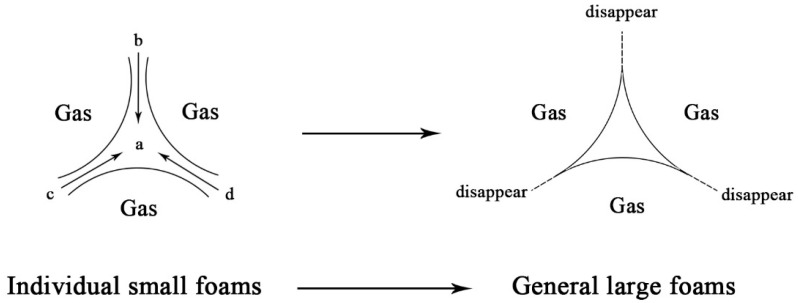
Consolidation within the foam.

A situation like the one shown in Figure 14 occurs for a standard round foam in the air. According to the general Equation (5) of the Young–Laplace equation, in the absence of other conditions, the large radius of curvature will discharge to the small radius of curvature due to the pressure difference. b, c, and d are located at the junction of two foams where the radius of curvature tends to infinity, while point a is at the junction of three foams whose radius of curvature is related to the thickness of the liquid film. The liquid film at points b, c, and d will drain towards point a, eventually leading to a rupture of the liquid film of the foam and forming a combined large foam.

(5) $$\Delta p=\gamma \left(\frac{1}{R_{1}}+\frac{1}{R_{2}}\right)$$

The effect of foaming agent types on the properties of the foam can be summarized in two points: Firstly, it affects the diameter of the prepared foam. Secondly, it affects the surface tension of the foam. From Equation (5), it can be judged that the foam of Type-Q is more stable in air, on the one hand. On the other hand, it forms a smaller diameter foam because its surface tension is less than that of Type-H and Type-S. The previous experimental data shows that the internal conditions of the QD80 group of foamed lightweight clay slurry are pretty different from those of SD100 and HD60. It can be assumed that due to the small diameter and low surface tension of most of the QD80 foams, it is difficult to achieve uniform dispersion during mixing with the slurry, and some of the foams still agglomerate to form a “foam isolation zone”. In this zone, the slurry is smaller and foamy, which makes it easier for the foam to merge and form more oversized foams. This also explains the tendency for the pore size distribution of the prepared FLS to be bipolar after hardening, despite the small diameter of the QD80 group foam in the air. However, although the HD60 and SD100 groups are relatively similar in terms of pore size distribution, there is still a difference in the compressive strength of FLS specimens, which may be related to the composition of the foaming agent itself or the charge carried by the liquid film, for which there is not yet a reasonable explanation.

Therefore, it can be concluded that the influence of foaming agent types on the FLS is a complex situation. Judging the foaming agent’s goodness and the prepared FLS’s quality is impossible simply from a few performances. However, one thing is clear: the foam prepared using the foaming agent is stable in the air but not necessarily in the FLS slurry.

In general, because manufacturers keep their technology secret, the internal composition of different foaming agents is not precise, and it is impossible to model the interaction between foam and slurry to establish the relationship between the three directly. However, using the same foaming agent, we can judge a positive correlation between the foam properties in the air, the properties in the FLS slurry, and the properties in FLS samples.

## 4. Conclusions

(1) The stability of foam in the air can be evaluated by using a 1h settling distance and bleeding rate. The stability of foam in the slurry can be evaluated by using the rate of increase in wet density after the defoaming test.
(2) The stability of foam in the air is related to the liquid film’s size and thickness. However, the stability of foam in the slurry is additionally associated with compatibility. For the same type of foaming agent, the stability of foam of different foam density in the air is the same as that in the slurry, with 50 kg/m^3^ as the best. For different types of foaming agents, the stability in the air is not the same as in the slurry for the same foam density. Experimental measurements are necessary to determine the stability of foaming agent types.
(3) Foams with a 40–60 kg/m^3^ density are irregularly polygonal in the air and return to their original round shape when entering the slurry. According to the Young–Laplace equation, foam with a larger diameter adsorbs more cementitious material particles on the surface during slurry incorporation and mixing, thus obtaining superior stability. Smaller-diameter foams will more readily undergo surface tension drainage and merge into more oversized-diameter foams.
(4) For the FLS with a design wet density of 600 kg/m^3^, a foam density of 50 kg/m^3^ gives better performance. After hardening, the samples prepared with foaming agent Types-S had the smallest average pore size of 299 μm and the highest compressive strength of 2.04 MPa at 28 d. The FLS prepared with the above three foaming agents met the technical specifications of the actual road-filling projects.

## Figures and Tables

**Table 1:** Physical properties of P·O 42.5 cement.

Density/(kg/m^3^)	Specific Surface Area/(m^2^/kg)	Soundness of Cement/mm	Setting Time/min		Flexural Strength/MPa		Compressive Strength/MPa	
			Initial	Final	3 Days	28 Days	3 Days	28 Days
3100	340	2	170	235	5.6	8.7	28.1	50.4

**Table 2:** Chemical composition of raw materials (wt%).

Material	CaO	SiO_2_	Al_2_O_3_	Fe_2_O_3_	MgO	SO_3_	K_2_O	Na_2_O	TiO_2_	LOI
PC	60.11	20.92	5.76	3.24	1.15	2.86	0.88	0.14	0.31	4.17
GBFS	39.92	31.23	14.12	0.78	7.34	2.23	0.61	0.72	0.76	−0.29
FA	0.44	57.64	21.49	6.52	1.77	0.37	3.42	0.12	0.93	6.85

**Table 3:** Mix ratios of FLS for different foam properties (kg/m^3^).

No.	PC	FA	GBFS	Water	Foam Properties		
					Types	Density	Dilution Factor
SF40	105	105	140	227.5	S	40	100
SF50	105	105	140	227.5	S	50	100
SF60	105	105	140	227.5	S	60	100
HD60	105	105	140	227.5	H	50	60
QD80	105	105	140	227.5	Q	50	80
SD100	105	105	140	227.5	S	50	100

**Table 4:** Performance of different foaming agents at different foam densities.

Item	Types of Foaming Agents								
	S (Dilution 100 Times)			H (Dilution 60 Times)			Q (Dilution 80 Times)		
Foam density (g/L)	40.5	49.2	60.3	42.2	50.2	60.0	41.4	51.2	60.2
Settlement (mm)	4.5	2	3	5	8	6	2	1	2
Bleeding rate (%)	70.19	72.66	76.72	68.69	76.84	82.17	58.00	59.82	63.19

**Table 5:** Parameters of the foam at different foam densities.

No.	Average Diameter (μm)	Average Liquid Film Thickness (μm)
SF40	167.12	22.99
SF50	236.28	38.59
SF60	199.21	23.62

**Table 6:** Parameters of foams with different foaming agents.

No.	Average Diameter (μm)	Average Liquid Film Thickness (μm)
SD100	236.28	38.59
HD60	147.69	21.27
QD80	121.75	22.54

**Table 7:** Rate of increase in wet density with different foam density.

Times	SF40		SF50		SF60	
	Measured Wet Density (kg/m^3^)	Rate of Increase (%)	Measured Wet Density (kg/m^3^)	Rate of Increase (%)	Measured Wet Density (kg/m^3^)	Rate of Increase (%)
0	605	0	607	0	611	0
1	614	1.38	612	0.82	632	3.44
2	626	3.36	623	2.64	658	7.69
3	641	5.83	631	3.95	681	11.46
4	654	7.98	645	6.26	699	14.40
5	667	10.13	656	8.07	712	16.53
6	679	12.11	662	9.06	736	20.46

**Table 8:** Rate of increase in wet density with different types of foaming agents.

Times	SD100		QD80		HD60	
	Measured Wet Density (kg/m^3^)	Rate of Increase (%)	Measured Wet Density (kg/m^3^)	Rate of Increase (%)	Measured Wet Density (kg/m^3^)	Rate of Increase (%)
0	607	0	609	0	625	0
1	612	0.82	612	0.38	637	1.81
2	623	2.64	619	1.53	651	4.05
3	631	3.95	635	4.16	668	6.77
4	645	6.26	668	9.57	683	9.16
5	656	8.07	692	13.50	698	11.56
6	662	9.06	727	19.25	713	13.96

**Table 9:** Pore size parameters of the different groups of FLS.

No.	Average Diameter (μm)	Mean Deviation (μ)	Standard Deviation (σ)
SF40	365.64	302	172
SF50	299.37	328	101
SF60	324.52	350	131

**Table 10:** Pore size parameters of FLS under different foaming agents.

No.	Average Diameter (μm)	Mean Deviation (μ)	Standard Deviation (σ)
SD100	299.37	325	101
HD60	307.38	328	104
QD80	343.91	350	283

## Data Availability

The data that support the findings of this study are available from the corresponding author upon reasonable request.
